# TBP Dynamics during Mouse Oocyte Meiotic Maturation and Early Embryo Development

**DOI:** 10.1371/journal.pone.0055425

**Published:** 2013-01-31

**Authors:** Shao-Chen Sun, Xu-Guang Wang, Xue-Shan Ma, Xian-Ju Huang, Juan Li, Hong-Lin Liu

**Affiliations:** College of Animal Science and Technology, Nanjing Agricultural University, Nanjing, China; Institute of Zoology, Chinese Academy of Sciences, China

## Abstract

To maintain cell lineage, cells develop a mechanism which can transmit the gene activity information to the daughter cells. In mitosis, TBP (TATA-binding protein), a transcription factor which belongs to TFIID was associated with M phase chromosomes and was proved to be a bookmark for cellular memory. Although previous work showed that TBP was dispensable for mouse oocyte maturation and early embryo development, exogenous TBP protein was detected in the nuclear of oocytes and early embryos. It is still unknown whether exogenous TBP can associate with condensed chromosomes during meiosis and mouse early embryo development. In present study by the injection of GFP-tagged TBP mRNA we for the first time investigated TBP dynamics in mouse early embryos and confirmed its localization pattern in oocytes. The exogenous TBP enriched at germinal vesicle at GV stage but disappeared from the chromosomes after GVBD. Moreover, exogenous TBP was still dispersed from the chromosomes of somatic donor nuclear in oocytes by nuclear transfer (NT), further proving that oocyte has some mechanism to remove TBP. During mouse embryo development, the exogenous TBP was removed from the chromosomes of M phase zygotes, but was found to express weakly at the M phase of 2-cell. Moreover, in the blastocyst TBP was also detected at the M phase chromosomes. Overexpression of TBP caused the failure of oocyte maturation and embryo development. Our results supported the idea that TBP might be a marker for transmitting cellular memory to daughter cells.

## Introduction

To maintain cell lineage cells develop a mechanism called gene bookmarking, which can transmit the gene activity information to the daughter cells [1]. This mechanism could remember the active genes before the cells enter M phase, and transmits the cellular memory to the daughter cells [2]. The transcription patterns can be faithfully established at G1 phase in an epigenetic manner, furthur ensures the daughter cells have the same pattern of gene expression compared with mother cell. However, till now the understanding about the gene bookmarking is still poor [3].

During the cell division process, the preinitiation complex is critical for the initiation of general gene transcription. This complex includes RNA polymerase II (Pol II) and the general transcription factors TFIIA, B, D, E, F and H [4]. While TBP (TATA-binding protein) is the core component of promoter recognition factor TFIID [5]. TBP could regulate the binding of TFIID to the promoters, while TBP-like factor (TLF/TRF2/TRP) and TBP2 (TRF3/TBPL2) are shown to mediate Pol II transcription [6], [7]. In the mitosis of somatic cells, TBP is proved to be a gene bookmarking factor [8], [9]. Evidence from the localization pattern of GFP-tagged-TBP showed that TFIID remains tightly at the condensed chromosomes in mitotic cells [10], and TBP also remain associated with promoters of genes that are active in cells. Moreover, TBP could recruits PP2A and interacts with condensin for preserving the memory of gene activity to the daughter cells [11].

After fertilization, the first cleavage of mouse embryo was regulated by the maternal components which were from the oocyte [12]. In late 2-cell stage, zygotic genome activation (ZGA) occurred and the embryo underwent the cellular reprogramming [13]. The transcription reprogramming is important for the regulation of the genome-wide epigenetic state during embryo development and the mechanisms of induced pluripotency [14], [15]. Previous work showed that in mouse and Xenopus oocytes, TBP is dispensable and TBP2 is the major factor for the germ cells, since TBP2 mRNA has been detected specifically in the oocyte and TBP2 protein localizes in the nucleus [16], [17]. TBP and TBP2 could mediate transcription by RNA polymerases in oocytes [17], [18]. Although TBP is dispensable for oocyte maturation and early embryo development, TBP protein exists in the nucleus of oocytes and zygotes [19]. The nuclear concentration of TBP decreased during oocyte growth, and increased in a time-dependent fashion following fertilization. However, the results examined by immunofluorescence method may be due to the defects that the antibody could not bind the chromosomes. Moreover, the localization pattern of TBP during mouse early embryo development is still unknown.

In present study we produced GFP tagged TBP mRNA and injected into mouse oocytes and zygotes to examine the localization pattern of TBP. Our results further confirmed that TBP was removed from the chromosomes by some unknown mechanism in oocytes in an exogenous TBP approach, more importantly we found that TBP expressed at M phase of blastomere in embryos from 2-cell stage. Overexpression of TBP caused the failure of oocyte maturation and embryo development. Therefore, TBP may be not only a transcription factor but also a marker for the maintenance of cellular memory in mouse embryo.

## Results

### Exogenous GFP-TBP disperses from chromosomes during mouse oocyte maturation

**Figure 1:**
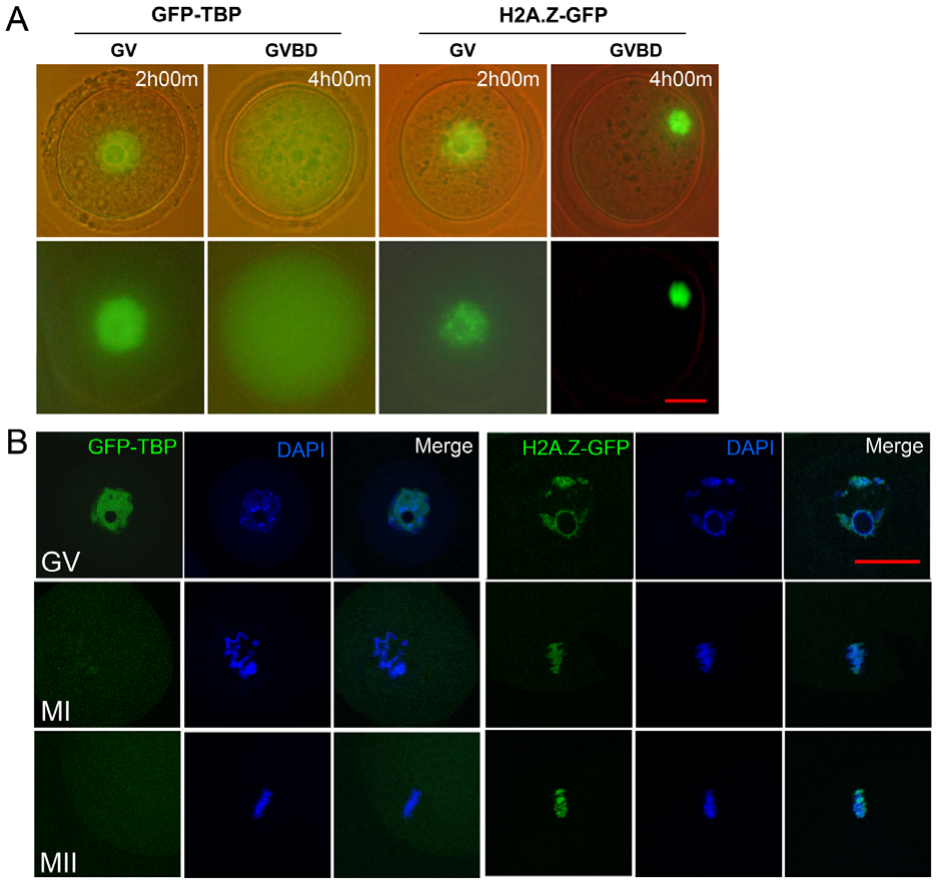
Localization pattern of GFP-TBP in mouse oocytes. (**A**) Time lapse results of GFP-TBP expression during mouse oocyte meiotic maturation. GFP-TBP accumulated in the germinal vesicle after 2 h culture with IBMX but degraded at 4 h with fresh medium, while the control H2A.Z-GFP localized at the chromosomes after 4 h culture. (**B**) Confocal microscopy results of GFP-TBP localization during mouse oocyte meiotic maturation. In the GV stage, GFP-TBP accumulated in the germinal vesicle; in the MI and MII stage, no GFP-TBP signals were found at the chromosomes of oocytes. The control H2A.Z-GFP localized at the chromosomes of MI and MII stage oocytes. Bar = 20 µm.

We produced GFP-TBP mRNA and microinjected into the GV stage oocytes and zygotes respectively. After 2 h incubation, the exogenous GFP-TBP successfully expressed and was detected in the germinal vesicle of oocyte and the nucleus of zygote, which is similar with somatic cells (Figure S1). Then we examined the dynamic expression and localization pattern of GFP-TBP during mouse oocyte maturation. Time lapse microscopy results showed that although the GFP-TBP accumulated in the germinal vesicle of oocyte, GFP-TBP still dispersed from the chromosomes after GVBD (germinal vesicle break down) (Figure 1A). Confocal microscopy results confirmed this, the results showed that in the MI and MII stage oocyte, there is no expression of exogenous GFP-TBP at the chromosomes (Figure 1B). To confirm the specificity of GFP-TBP mRNA and the dynamic expression pattern, we also injected H2A.Z-GFP mRNA into the oocyte, and H2A.Z-GFP showed normal localization pattern. H2A.Z-GFP expressed in the germinal vesicle of GV oocyte and at the chromosomes after GVBD (Figure 1A, B).

**Figure 2:**
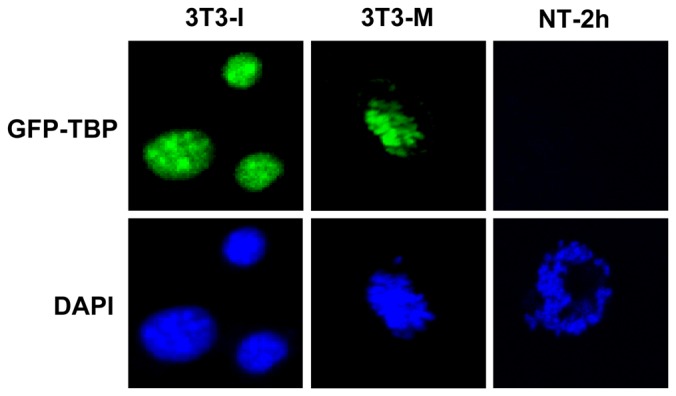
Localization of GFP-TBP in 3T3 cells and NT oocytes. GFP-TBP localized at the nucleus of interphase 3T3 cells and chromosomes of M phase 3T3 cells. However, no GFP-TBP signal was found at the chromosomes of NT oocytes.

To confirm this, we also transfected 3T3 cells with GFP-TBP and examined the expression of GFP-TBP in 3T3 cells. As shown in Figure 2, exogenous GFP-TBP signals were detected in both interphase and mitotic (M) phase cells, which is similar with previous work. However, there was no GFP-TBP expression at the chromosomes (which comes from 3T3 cell) of nuclear transfer (NT) oocyte (Figure 2). Therefore, GFP-TBP was specifically degraded in the mouse oocytes.

### Localization pattern of exogenous GFP-TBP during mouse embryo development

**Figure 3:**
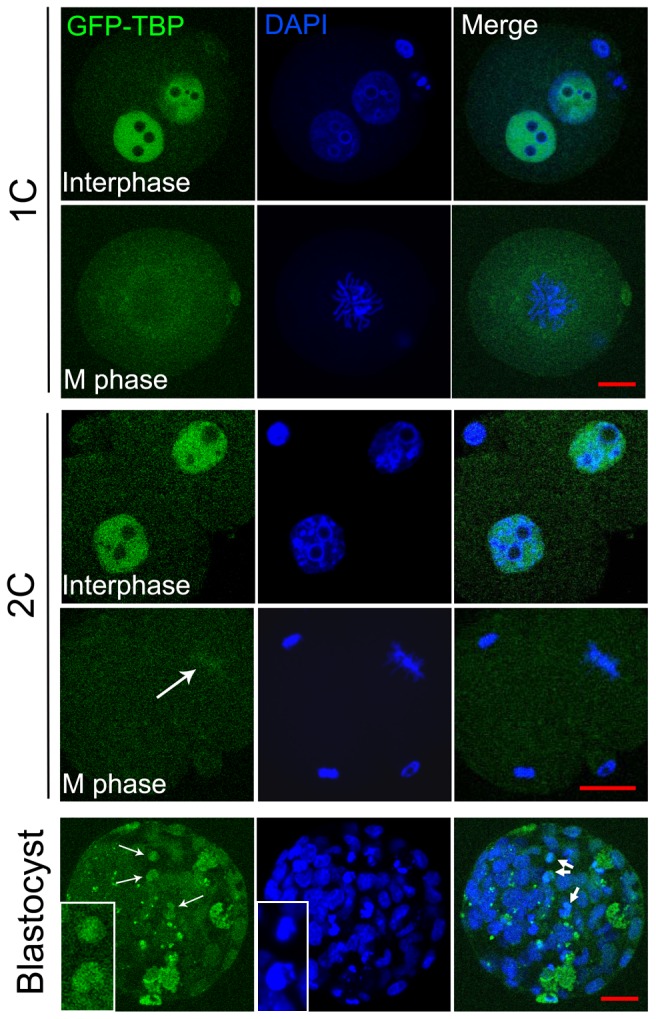
Localization of GFP-TBP in mouse embryos. GFP-TBP signals were found at the nucleus of interphase of the zygotes and 2 cell embryos. In the M phase of zygotes, there was no GFP-TBP expression; In the M phase of 2-cell embryos, a weak GFP-TBP signal was found at the chromosomes; in the blastocyst, GFP-TBP was detected at the chromosomes of M phase blastomeres. Arrow shows the chromosome localization of exogenous TBP expression. Bar = 20 µm.

We then examined the localization pattern of GFP-TBP during mouse embryo development. As shown in Figure 3, exogenous GFP-TBP could be detected in the nucleus of zygotes but still was removed from the chromosomes at M phase zygotes. In the 2-cell embryos, TBP localized at the nucleus at the interphase, and different with zygotes, we found weak GFP-TBP signal at the chromosomes of M phase 2-cell embryos. And interestingly, GFP-TBP also expressed largely at the chromosomes of M phase blastomeres in the blastocysts. While in the control group, different with GFP-TBP, H2A.Z-GFP expressed not only at the nucleus of zygotes and interphase 2-cell embryos, but also localized at the chromosomes of zygotes and M phase 2-cell embryos (Figure S2). Therefore, from 2-cell stage GFP-TBP might increase at the chromosomes of M phase mouse embryos.

### Overexpression of TBP inhibits oocyte maturation and embryo development

**Figure 4:**
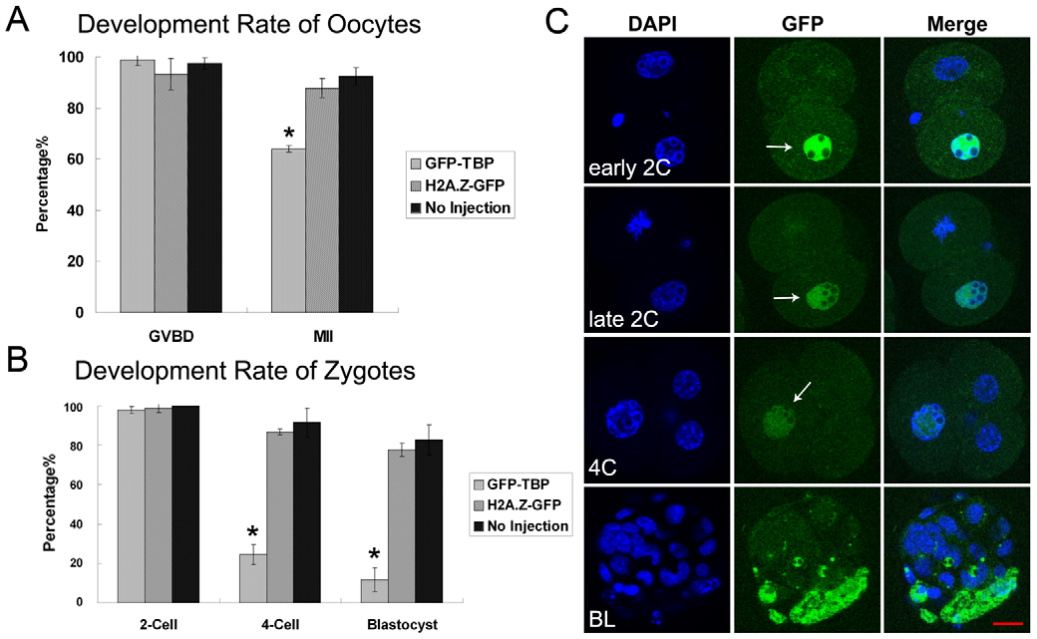
Effects of GFP-TBP overexpression on mouse oocyte maturation and embryo development. (**A**) Rates of GVBD oocytes and MII oocytes in GFP-TBP overexpression group. Overexpression of TBP had no effect on oocyte GVBD compared with control group, but rate of MII stage oocyte was significantly lower that the control group. (**B**) Rates of 2-cell embryo, 4-cell embryo and blastocyst in GFP-TBP overexpression group. Overexpression of TBP had no effect on the first cleavage, but the rate of 4-cell was significantly lower than the control group. Moreover, the rate of blastocyst was also significantly lower than the control group. (**C**) The embryo development after the injection of GFP-TBP mRNA into one cell of 2-cell embryos. The cell which was injected with GFP-TBP mRNA failed to undergo cell division. The cell with no injection showed normal cell division. Arrow shows the nucleus localization of exogenous TBP expression. *, significantly different (p<0.05). Bar = 20 µm.

We also examined the effects of TBP overexpression on oocyte maturation and embryo development. As shown in Figure 4A, overexpression of TBP had no effect on oocyte GVBD compared with control group (98.8%±2.1%, n = 78; H2A.Z-GFP 93.3%±6.1%, n = 73; No injection, 97.5%±2.3%, n = 79) (p>0.1), but rate of MII stage oocyte was significantly lower that the control group (64.1%±1.2%; H2A.Z-GFP 87.8%±3.1%; No injection, 92.5%±3.3%) (p<0.05). For the embryo development, overexpression of TBP had no effect on the first cleavage (97.9%±1.9%, n = 94 vs H2A.Z-GFP 98.7%±2.1%, n = 76; No injection 100% n = 70) (p>0.1), but the rate of 4-cell was significantly lower than the control group (24.5%±5.1%, n = 94 vs H2A.Z-GFP 86.8%±1.5%, n = 76; No injection 91.4±7.5% n = 70) (p<0.05), moreover, the rate of blastocyst was also significantly lower than the control group (11.7%±6.1%, n = 94 vs H2A.Z-GFP 77.6%±3.2%, n = 76; No injection 82.9±7.5% n = 70) (p<0.05) (Figure 4B). To confirm this, we also injected GFP-TBP mRNA into one cell of early 2-cell embryos. As shown in Figure 4C, the cell injected with GFP-TBP mRNA failed to undergo cytokinesis and went to apoptosis, while the cell with no GFP-TBP mRNA injection underwent normal cell division (Figure 4C). The results indicated that overexpression of TBP could inhibit the oocyte maturation and embryo development.

## Discussion

In present study we injected GFP-tagged TBP mRNA into mouse oocytes and zygotes to examine the expression pattern of TBP. We found that even the exogenous GFP-TBP was still dispersed in mouse oocyte and zygote, but TBP was found to localize at the chromosomes of the M phase blastomere from 2-cell stage during embryo development. Overexpression of TBP inhibited oocyte maturation and embryo cleavage. Since the reprogramming occurs after fertilization, our results indicated that TBP might be a marker for the cellular memory during mouse embryo development.

It is shown that TBP is dispensable for oocytes, instead, TBP homologue TBP2 is the major factor for the regulation of germ cells. Moreover, TBP2 together with TBP could mediate transcription by RNA polymerases in oocytes [16], [17]. The finding of TBP protein expression in the GV oocytes disagrees with results achieved in previous studies in mouse and Xenopus oocytes [16], [17], where only the TBP2 has been evidenced at this stage of development. Moreover, these studies also showed that, when artificially provided, TBP can substitute for TBP2 in the oocytes core transcription machinery, as the two proteins share 95% sequence identity in the core domain. On the other hand, another study evidenced TBP protein in GV oocytes by immunofluorescence, resulting in a controversy [19]. Our results, which examined the localization of exogenous TBP, showed this expression pattern: although exogenous TBP could accumulate in the germinal vesicle, it was still disappeared from the chromosomes after oocyte GVBD, indicating that there was some unknown mechanism to regulate this process. To further examine whether this mechanism is specific for oocyte, we also examined the localization pattern of GFP-TBP in 3T3 cells, and similar results were shown compared with previous work [10], GFP-TBP localized at the chromosomes at M phase cells, which is the feature of TBP as gene bookmarking factor [1]. Furthermore, when a nucleus of a 3T3 cell expressed GFP-TBP was transmitted to an enucleated oocyte, GFP-TBP was still dispersed from the chromosomes of the donor (3T3) cell; which suggest that a specific mechanism that removes TBP from the chromosomes exist in the oocyte.

In early embryos the maternal TBP mRNA translation is activated to produce an abundant pool of TBP before zygotic gene activation (ZGA), indicating that TBP may be involved into the embryo development [7], [19], [20]. However, it was shown that TBP was dispensable for mouse embryo development. We then explored the localization pattern of TBP at M phase blastomere during embryo cleavage. Our results showed that similar with oocyte, TBP was still dispersed from the chromosomes during the first cleavage of embryo. Since the maternal components still rule this process, together with the degradation of TBP in oocyte, it is reasonable to hypothesize that it is the maternal components which regulate the localization of TBP to the chromosomes. However, the inhibition mechanism might be unlocked at 2-cell stage, since we detected the weak signal at the chromosomes in the M phase 2-cell embryos. The reprogramming occured after fertilization and zygotic gene activation (ZGA) initiated at the late 2-cell stage, which is important for the regulation of the genome-wide epigenetic state and the mechanism of induced pluripotency. To prove our hypothesis, we also examined the few blastocysts we got after GFP-TBP mRNA microinjection, and we found at GFP-TBP expressed strongly at the chromosomes of M phase blastomeres. TBP was shown to accumulate at the chromosomes of M phase somatic cells, and this was the feature of TBP as gene bookmarking factor. Therefore, the expression of exogenous TBP at the chromosomes of 2-cell embryo indicated that TBP might also be a cellular memory marker for the maintenance of genome-wide reprogramming during embryo development.

Overexpression of GFP-TBP caused the failure of mouse oocyte maturation and embryo development, indicating that the repression of TBP activity in the oocytes and zygotes is necessary for the normal development. Our results indirectly confirmed the previous work which showed that TBP is dispensable for oocyte maturation [16], [17]. However, it is still unclear why embryo development is affected after TBP overexpression, since depletion of TBP has no effect on the ovulation and fertilization [17]. One hypothesis is that exogenous mRNA may have toxic effect on the cell. Moreover, the fact that overexpression of TBP leads to a failure in oocyte maturation and embryo development may support the hypothesis that TBP is replacing ‘natural’ TBP2.

In conclusion, our results confirmed the remove of TBP in mouse oocytes, and showed that the expression of TBP at the chromosomes at M phase blastomeres from 2-cell stage during mouse embryo development. It is necessary to remove that TBP exogenously added from chromosomes for oocytes and maternal-controlled zygotes, since overexpression of TBP caused aberrant oocyte maturation and embryo development. We concluded that TBP may be also a gene bookmarking factor to maintain cellular memory after the reprogramming in mouse embryos.

## Materials and Methods

### Ethic statement

Animal care and use were conducted in accordance with the Animal Research Institute Committee guidelines of Nanjing Agricultural University, China. Mice were housed in a temperature-controlled room with proper darkness-light cycles, fed with a regular diet, and maintained under the care of the Laboratory Animal Unit, Nanjing Agricultural University. The mice were killed by cervical dislocation. This study was specifically approved by the Committee of Animal Research Institute, Nanjing Agricultural University.

### Oocyte and zygote collection

Germinal vesicle-intact oocytes were collected from the ovaries of 6- to 8-week-old BDF1 mice and were cultured in α-MEM medium (Gbico) under paraffin oil at 37°C, 5% CO_2_. Oocytes were then collected for microinjection. For zygotes collection, after injection PMSG for 48 h, the 6- to 8-week-old BDF1 mice were injected with hCG and mated with male mouse immediately. Zygotes were collected after 18 h and were cultured in α-MEM medium (Gbico) under paraffin oil at 37°C, 5% CO_2_.

### GFP-TBP and H2A.Z GFP mRNA production

pEGFP-TBP-C1 vector was a gift from Professor Aoki at University of Tokyo, Japan. H2A.Z mRNA and first strand cDNA were generated from the mouse liver. After extracting the PCR product with gel extraction kit (Promega), the H2A.Z cDNA was subcloned to TOPOII vector and transfected into Top10 E.coli. We then extracted plasmid (Tiangen Kit) and acquired the correct sequence by double enzyme cut (*EcoR*I*,Bgl*II). The H2A.Z cDNA was subcloned into pEGFP-N1 vector for *in vitro* transcription. The pH2A.Z-GFP-N1 plasmid was linearized by *Hind*III and purified by gel extract kit (Promega). T7 message machine (Ambion) was used for producing capped mRNA, and then mRNA was purified by RNeasy cleanup kit (Qiagen).

### GFP-TBP and H2A.Z GFP mRNA microinjection

The microinjection was performed as previous described [21], [22]. Approximately 5–10 pl of GFP-TBP and H2A.Z GFP mRNA was microinjected into the cytoplasm of a fully-grown GV oocyte or zygote using an Narishige microinjector (Narishige Inc., Sea Cliff, NY) with a Nikon Diaphot ECLIPSE TE300 inverted microscope (Nikon UK Ltd., Kingston upon Thames, Surrey, UK) equipped with a Narishige MM0-202N hydraulic three-dimensional micromanipulator (Narishige Inc., Sea Cliff, NY). After injection, the oocytes were cultured in α-MEM medium containing 0.2 µg/ml IBMX for 3 h, and then washed five times in fresh α-MEM medium. The zygotes were cultured in fresh α-MEM medium. The ooctyes with GFP-fluorescence were cultured in α-MEM medium without IBMX under paraffin oil at 37°C in an atmosphere of 5% CO_2_ in air.

### Nuclear transfer

The transfection of NIH 3T3 cells with GFP-TBP mRNA and the enucleation of oocytes was conducted as described previously [23]. The nuclei of NIH 3T3 cells were introduced into the enucleated oocytes by electrofusion, using a DC pulse of 1,500 V/cm for 20 µs in 300 mM mannitol that contained 0.1 mM MgSO_4_, 0.1 mg/ml polyvinyl alcohol, and 3 mg/ml bovine serum albumin.

### Time-lapse microscopy and confocal microscopy

Oocytes were incubated with α-MEM medium for imaging chromosome dynamics during oocyte maturation. Images were taken by a 20×/0.5 objective lens (Carl Zeiss, Germany) under a computer controlled video microscope (Carl Zeiss, Germany). We captured images every 20 min and the ZEN software (Carl Zeiss) was used to analyze the resulting video files.

For confocal microscopy, the samples were co-stained with DAPI for 10 min, and then examined with a confocal laser-scanning microscope (Zeiss LSM 700 META). At least 20 oocytes were examined for each group.

### Data analysis

At least three replicates were performed for each experiment. Statistical analyses were conducted using an analysis of variance (ANOVA) and differences between treatment groups were evaluated with Duncan's multiple comparison test. Data were expressed as mean ± SD and p<0.05 was considered to be statistically significant.

## Supporting Information

Figure S1 **Identification of the expression of GFP-TBP mRNA in oocytes and zygotes.** GFP-TBP successfully expressed in the germinal vesicle of oocytes and pronucleus of zygotes. The constructed GFP-TBP mRNA was also identified. Bar = 50 µm.
(TIF)

Figure S2 **Identification of the expression of H2A.Z-GFP in the interphase, M phase of zygotes and 2-cell embryos.** In the interphase, H2A.Z-GFP expressed in the nucleus; in the mitotic phase, H2A.Z-GFP expressed at the chromosomes. Zygote I: Zygote Interphase; Zygote M: Zygote Mitotic phase; 2-Cell I: 2-Cell Interphase; 2-Cell M: 2-Cell Mitotic phase; Bar = 50 µm.
(TIF)

## References

[1] Sarge KD, Park-Sarge OK (2005) Gene bookmarking: keeping the pages open. Trends Biochem Sci 30: 605–610. 16188444 10.1016/j.tibs.2005.09.004

[2] Gottesfeld JM, Forbes DJ (1997) Mitotic repression of the transcriptional machinery. Trends Biochem Sci 22: 197–202. 9204705 10.1016/s0968-0004(97)01045-1

[3] Michelotti EF, Sanford S, Levens D (1997) Marking of active genes on mitotic chromosomes. Nature 388: 895–899. 9278053 10.1038/42282

[4] Juven-Gershon T, Kadonaga JT (2009) Regulation of gene expression via the core promoter and the basal transcriptional machinery. Dev Biol 339: 225–229. 19682982 10.1016/j.ydbio.2009.08.009 PMC2830304

[5] Jones KA (2007) Transcription strategies in terminally differentiated cells: shaken to the core. Genes Dev 21: 2113–2117. 17785521 10.1101/gad.1598007

[6] Deato MD, Marr MT, Sottero T, Inouye C, Hu P, et al (2008) MyoD targets TAF3/TRF3 to activate myogenin transcription. Mol Cell 32: 96–105. 18851836 10.1016/j.molcel.2008.09.009 PMC2629732

[7] Bartfai R, Balduf C, Hilton T, Rathmann Y, Hadzhiev Y, et al (2004) TBP2, a vertebrate-specific member of the TBP family, is required in embryonic development of zebrafish. Curr Biol 14: 593–598. 15062100 10.1016/j.cub.2004.03.034

[8] Christova R, Oelgeschlager T (2002) Association of human TFIID-promoter complexes with silenced mitotic chromatin in vivo. Nat Cell Biol 4: 79–82. 11744923 10.1038/ncb733

[9] Segil N, Guermah M, Hoffmann A, Roeder RG, Heintz N (1996) Mitotic regulation of TFIID: inhibition of activator-dependent transcription and changes in subcellular localization. Genes Dev 10: 2389–2400. 8843192 10.1101/gad.10.19.2389

[10] Chen D, Hinkley CS, Henry RW, Huang S (2002) TBP dynamics in living human cells: constitutive association of TBP with mitotic chromosomes. Mol Biol Cell 13: 276–284. 11809839 10.1091/mbc.01-10-0523 PMC65088

[11] Xing H, Vanderford NL, Sarge KD (2008) The TBP-PP2A mitotic complex bookmarks genes by preventing condensin action. Nat Cell Biol 10: 1318–1323. 18931662 10.1038/ncb1790 PMC2577711

[12] Zhu S, Ma T, Li J, Ding S (2011) Recent advances in chemically induced reprogramming. Cell Cycle 10: 871–872. 21368574 10.4161/cc.10.6.15035 PMC3100870

[13] Simonsson S, Gurdon JB (2005) Changing cell fate by nuclear reprogramming. Cell Cycle 4: 513–515. 15753660 10.4161/cc.4.4.1581

[14] Miyamoto K, Pasque V, Gurdon JB (2011) Nuclear actin in transcriptional reprogramming by oocytes: are actin nucleators key players? Cell Cycle 10: 3040–3041. 21869611 10.4161/cc.10.18.16946 PMC3218615

[15] Grealish S, Jakobsson J, Parmar M (2011) Lineage reprogramming: a shortcut to generating functional neurons from fibroblasts. Cell Cycle 10: 3421–3422. 22067653 10.4161/cc.10.20.17691

[16] Akhtar W, Veenstra GJ (2009) TBP2 is a substitute for TBP in Xenopus oocyte transcription. BMC Biol 7: 45. 19650908 10.1186/1741-7007-7-45 PMC2731028

[17] Gazdag E, Santenard A, Ziegler-Birling C, Altobelli G, Poch O, et al (2009) TBP2 is essential for germ cell development by regulating transcription and chromatin condensation in the oocyte. Genes Dev 23: 2210–2223. 19759265 10.1101/gad.535209 PMC2751983

[18] Muller F, Tora L (2009) TBP2 is a general transcription factor specialized for female germ cells. J Biol 8: 97. 19951399 10.1186/jbiol196 PMC2804282

[19] Worrad DM, Ram PT, Schultz RM (1994) Regulation of gene expression in the mouse oocyte and early preimplantation embryo: developmental changes in Sp1 and TATA box-binding protein, TBP. Development 120: 2347–2357. 7925035 10.1242/dev.120.8.2347

[20] Gazdag E, Rajkovic A, Torres-Padilla ME, Tora L (2007) Analysis of TATA-binding protein 2 (TBP2) and TBP expression suggests different roles for the two proteins in regulation of gene expression during oogenesis and early mouse development. Reproduction 134: 51–62. 17641088 10.1530/REP-06-0337

[21] Sun SC, Wang ZB, Xu YN, Lee SE, Cui XS, et al (2011) Arp2/3 complex regulates asymmetric division and cytokinesis in mouse oocytes. PLoS One 6: e18392. 21494665 10.1371/journal.pone.0018392 PMC3072972

[22] Sun SC, Xu YN, Li YH, Lee SE, Jin YX, et al (2011) WAVE2 regulates meiotic spindle stability, peripheral positioning and polar body emission in mouse oocytes. Cell Cycle 10: 1853–1860. 21543895 10.4161/cc.10.11.15796

[23] Kim JM, Liu H, Tazaki M, Nagata M, Aoki F (2003) Changes in histone acetylation during mouse oocyte meiosis. J Cell Biol 162: 37–46. 12835313 10.1083/jcb.200303047 PMC2172711

