# Supplementary figures and images for: TBP Dynamics during Mouse Oocyte Meiotic Maturation and Early Embryo Development
Source: PLoS One. 2013 Jan 31;8(1):e55425. doi: 10.1371/journal.pone.0055425 (PMC3561223; doi:10.1371/journal.pone.0055425)

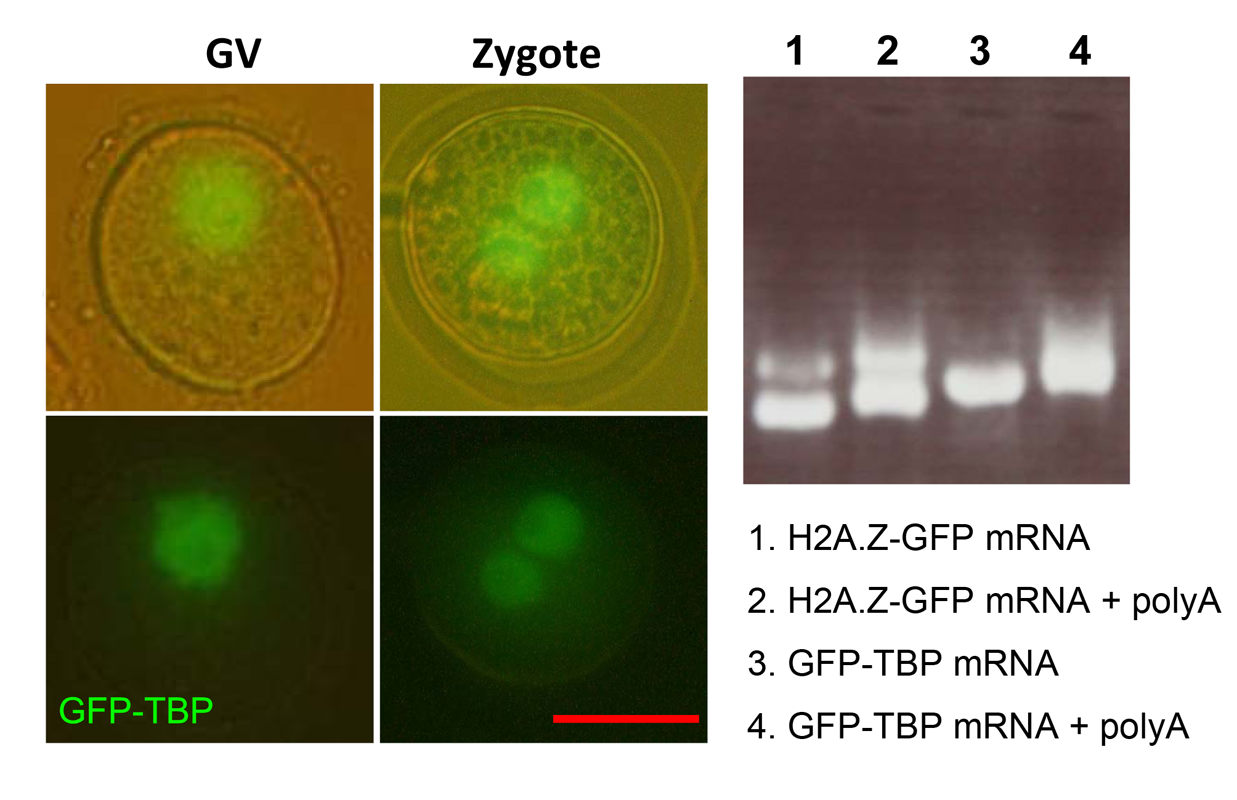

Supplement: Figure S1 — Identification of the expression of GFP-TBP mRNA in oocytes and zygotes. GFP-TBP successfully expressed in the germinal vesicle of oocytes and pronucleus of zygotes. The constructed GFP-TBP mRNA was also identified. Bar = 50 µm. (TIF) [file pone.0055425.s001.tif]

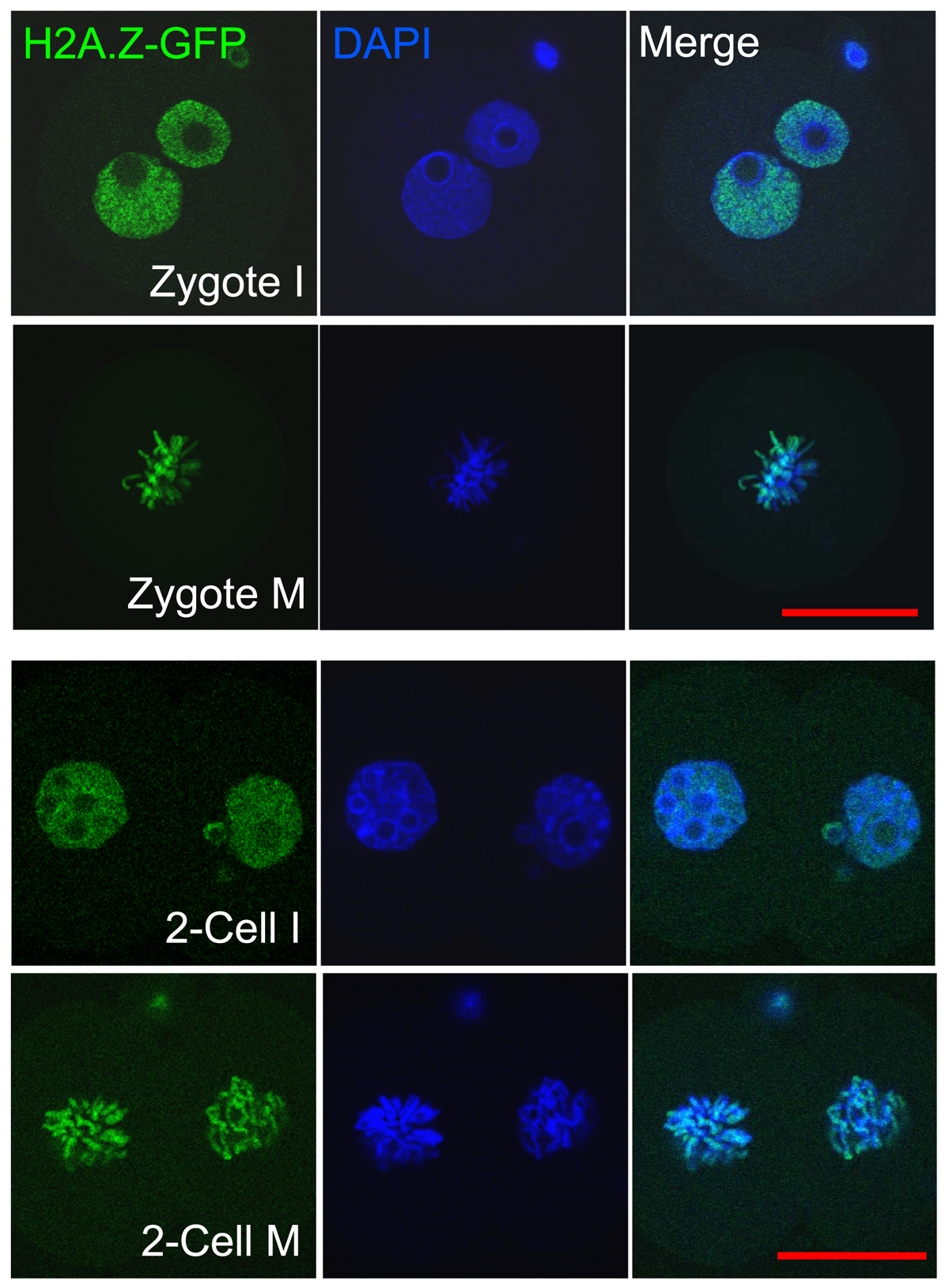

Supplement: Figure S2 — Identification of the expression of H2A.Z-GFP in the interphase, M phase of zygotes and 2-cell embryos. In the interphase, H2A.Z-GFP expressed in the nucleus; in the mitotic phase, H2A.Z-GFP expressed at the chromosomes. Zygote I: Zygote Interphase; Zygote M: Zygote Mitotic phase; 2-Cell I: 2-Cell Interphase; 2-Cell M: 2-Cell Mitotic phase; Bar = 50 µm. (TIF) [file pone.0055425.s002.tif]
